# Supplementary material for: CRISPR/Cas12a technology combined with immunochromatographic strips for portable detection of African swine fever virus
Source: Commun Biol. 2020 Feb 11;3:62. doi: 10.1038/s42003-020-0796-5 (PMC7012833; doi:10.1038/s42003-020-0796-5)
Supplement: Supplementary file 1 — Description of additional supplementary files [file 42003_2020_796_MOESM1_ESM.docx]

**Description of additional supplementary files**

**Supplementary Data 1.** Source data used to plot as follows:

Figure 1b, c, d, e

Figure 2c, e

Figure 3b, d, f

Figure 4c, d
