# Supplementary material for: CRISPR/Cas12a technology combined with immunochromatographic strips for portable detection of African swine fever virus
Source: Commun Biol. 2020 Feb 11;3:62. doi: 10.1038/s42003-020-0796-5 (PMC7012833; doi:10.1038/s42003-020-0796-5)
Supplement: Supplementary file 3 — Supplemental Information [file 42003_2020_796_MOESM3_ESM.pdf]

Supplementary Figures

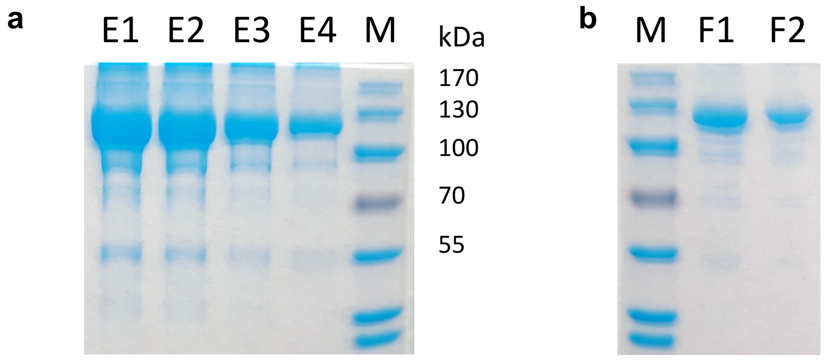

Supplementary Figure 1. Purification of LbCas12a protein. SDS-PAGE gel of all LbCas12 samples purified using Ni-NTA resin (**a**) and FPLC (**b**).

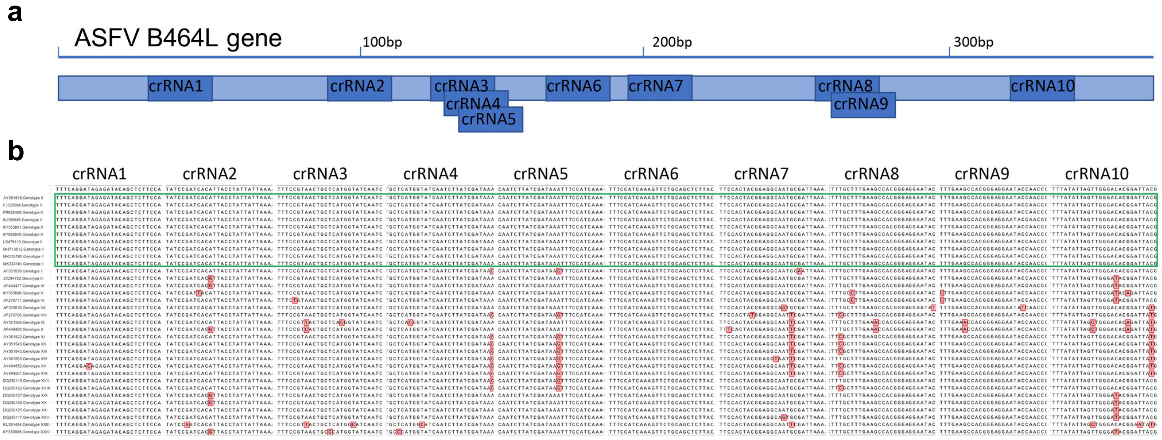

Supplementary Figure 2. Design of specific crRNAs for ASFV detection targeting B646L gene. (**a**) Ten crRNAs targeting B646L gene selected for ASFV detection. (**b**) Sequence alignment of B646L genes from 24 ASFV genotypes targeted by crRNAs. The nucleotide variants from the consensus sequence were highlighted with red color.

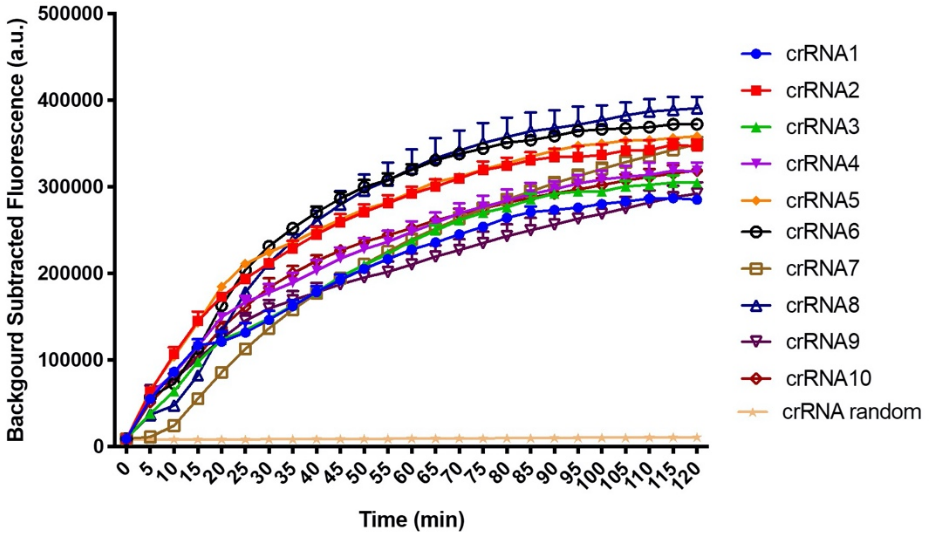

Supplementary Figure 3. Time course of ten crRNAs induced CRISPR/Cas12a reaction with ASFV B646L gene.

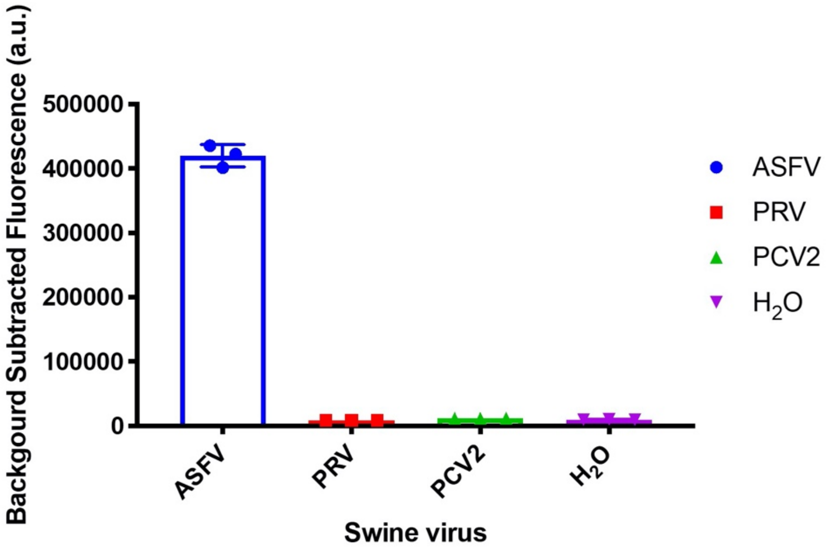

Supplementary Figure 4. Specificity of CRISPR/Cas12a detection of ASFV.

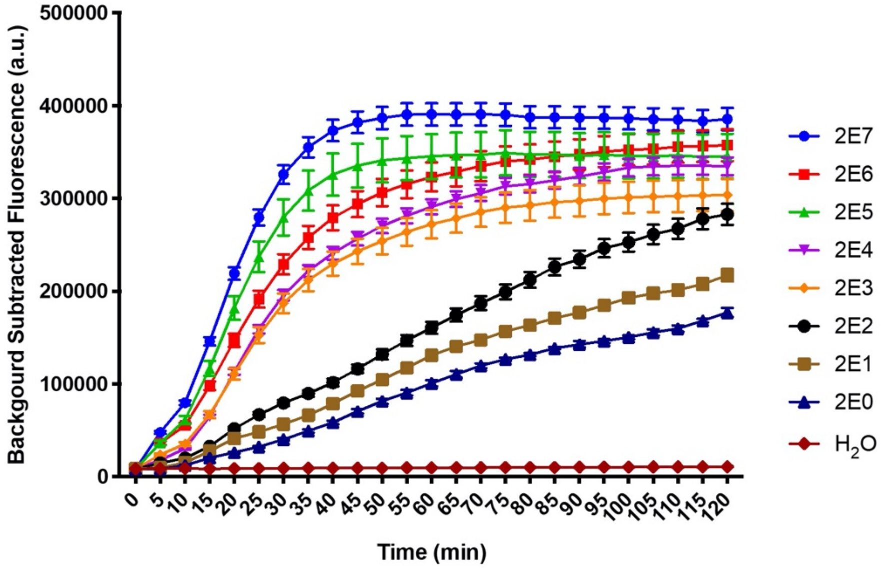

Supplementary Figure 5. Sensitivity of CRISPR/Cas12a detection of ASFV B646L gene combined with RAA application in 2-hour time course.

a

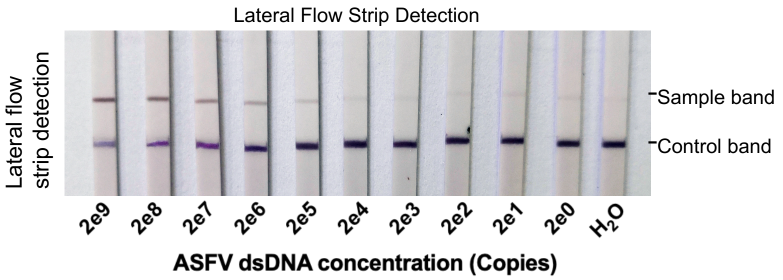

b

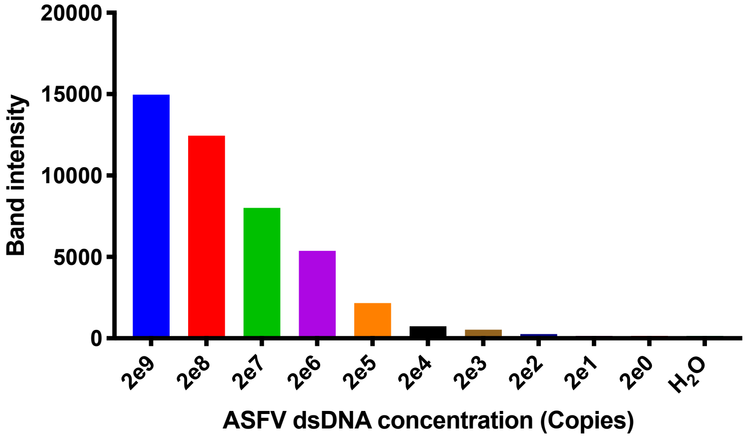

Supplementary Figure 6. Sensitivity of ASFV detection by CRSPR-Cas12a lateral flow detection. (**a**) Sensitivity of CRISPR/Cas12a-LFD detection of the synthetic AFSV DNA. (**b**) Quantitation of band intensity from detection in (**a**).

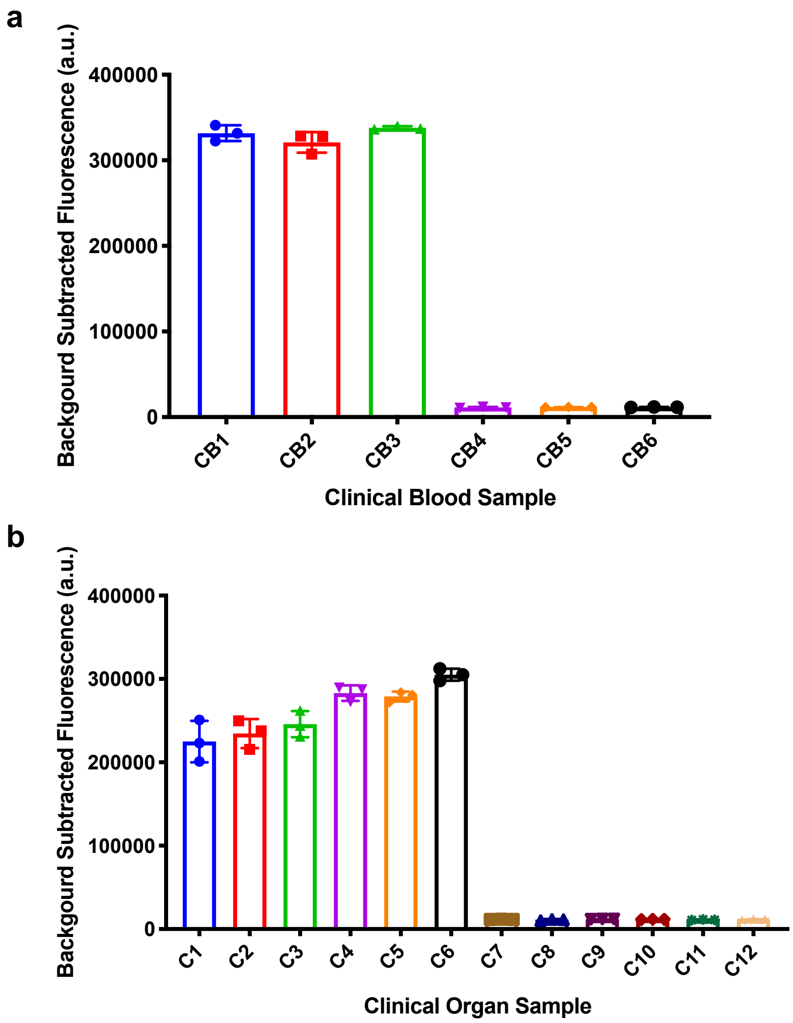

Supplementary Figure 7. Detection of ASFV DNA in clinical blood samples (**a**) and organ samples (**b**) with CRISPR-Cas12a fluorescence assay.

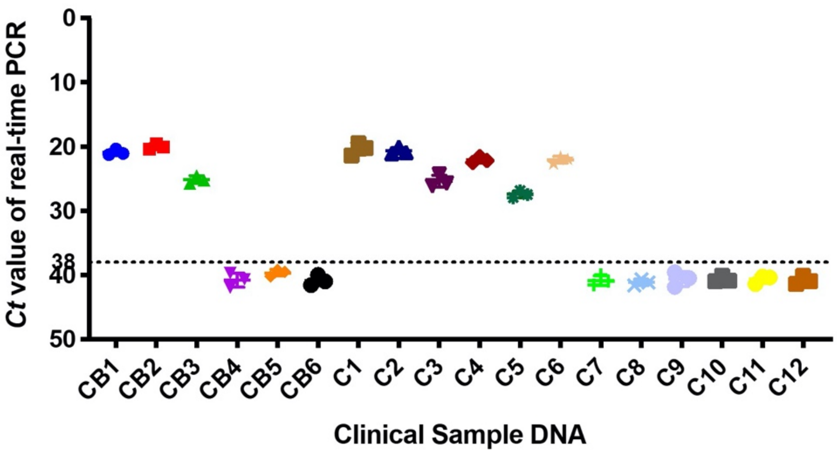

Supplementary Figure 8. Detection of ASFV DNA in clinical blood samples and organ samples by Real-time PCR. When Ct value below 38, the sample is ASFV positive.

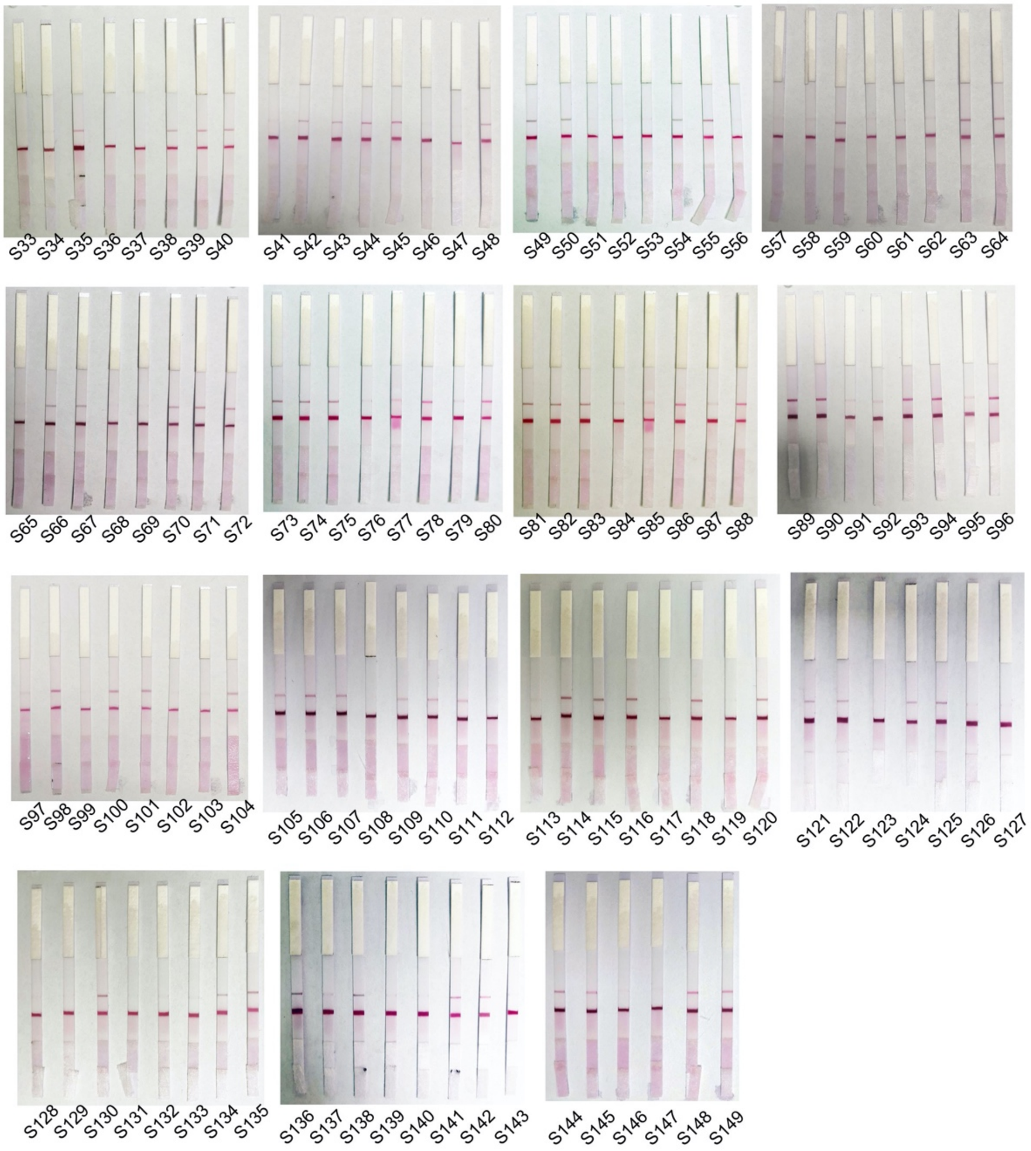

Supplementary Figure 9. Detection of ASFV DNA in 117 blood samples using CRISPR/Cas12a-LFD. The sample band and control band on the lateral strip were marked with arrows.

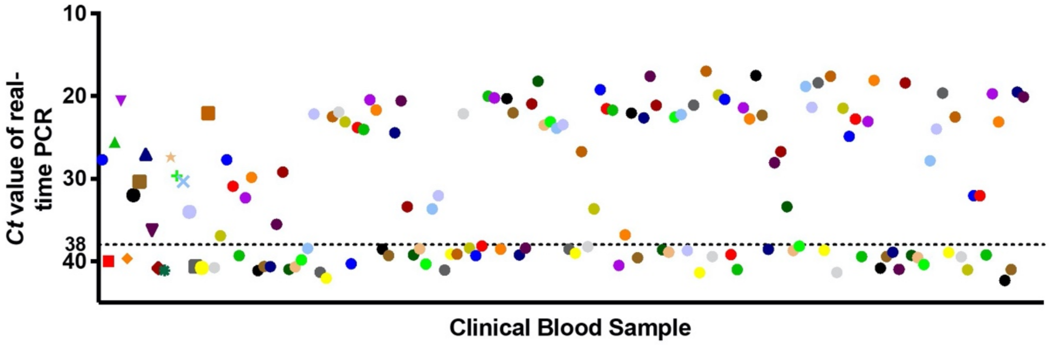

Supplementary Figure 10. Detection of ASFV DNA in 149 blood samples by Real-time PCR.

When Ct value below 38, the sample is ASFV positive.

Supplementary Tables

Supplementary Table 1. Primers used in this study

Primer name	Primer sequence (5' to 3')
cLbCas12a-F1	TACCATGGGTCCGAAAAAGAAACGC
cLbCas12a-R1	TTAGCAGCCGGATCTCAGTGGTGGT
LB-sgRNA-T7-IVF-F1	ACGGGAGGTACTTGGAGCGG
LB-sgRNA-T7-IVF-R1	TAGTGAGACGTGCGGCTTCCGTTT
p72-RAA-F1	TATGCAGCCCACTCACCACGCAGAGATAAG
p72-RAA-R1	CCGTTCTGAAGAAGAAGAAGCCGTAGTGATAGAC
ASFV-p72-F1	GATTGGCACAAGTTCGGACATGTT
ASFV-p72-R1	TTAGGTACTGTAACGCAGCACAGC
crRNA1-F1	AGATAGGATAGAGATACAGCTCTTCCA
crRNA1-R1	AAAATGGAAGAGCTGTATCTCTATCCT
crRNA2-F1	AGATATAATAGGTAATGTGATCGGATA
crRNA2-R1	AAAATATCCGATCACATTACCTATTAT
crRNA3-F1	AGATCGTAACTGCTCATGGTATCAATC
crRNA3-R1	AAAAGATTGATACCATGAGCAGTTACG
crRNA4-F1	AGATTCGATAAGATTGATACCATGAGC
crRNA4-R1	AAAAGCTCATGGTATCAATCTTATCGA
crRNA5-F1	AGATATGGAAATTTATCGATAAGATTG
crRNA5-R1	AAAAATGGAAATTTATCGATAAGATTG
crRNA6-F1	AGATCATCAAAGTTCTGCAGCTCTTAC
crRNA6-R1	AAAAGTAAGAGCTGCAGAACTTTGATG
crRNA7-F1	AGATATCGCATTGCCTCCGTAGTGGAA
crRNA7-R1	AAAATTCCACTACGGAGGCAATGCGAT
crRNA8-F1	AGATCTTTGAAGCCACGGGAGGAATAC
crRNA8-R1	AAAAGTATTCCTCCCGTGGCTTCAAAG
crRNA9-F1	AGATAAGCCACGGGAGGAATACCAACC
crRNA9-R1	AAAAGGTTGGTATTCCTCCCGTGGCTT
crRNA10-F1	AGATTATTAGTTGGGACACGGATTACG
crRNA10-R1	AAAACGTAATCCGTGTCCCAACTAATA

Supplementary Table 2. crRNAs targeting ASFV B646L gene

crRNA name	crRNA sequence with PAM (5' to 3')	Direction
crRNA1	*TTTC*AGGATAGAGATACAGCTCTTCCA	+
crRNA2	*TTTA*ATAATAGGTAATGTGATCGGATA	-
crRNA3	*TTTC*CGTAACTGCTCATGGTATCAATC	+
crRNA4	*TTTA*TCGATAAGATTGATACCATGAGC	-
crRNA5	*TTTG*ATGGAAATTTATCGATAAGATTG	-
crRNA6	*TTTC*CATCAAAGTTCTGCAGCTCTTAC	+
crRNA7	*TTTA*ATCGCATTGCCTCCGTAGTGGAA	-
crRNA8	*TTTG*CTTTGAAGCCACGGGAGGAATAC	+
crRNA9	*TTTG*AAGCCACGGGAGGAATACCAACC	+
crRNA10	*TTTA*TATTAGTTGGGACACGGATTACG	+

Note: The PAM of CRISPR/Cas12a recognize in *italic*.
